# “Prevalence, outcome and factors associated with dysglycemia among critically ill children presenting to Fort Portal Regional Referral Hospital: A cross sectional study”

**DOI:** 10.21203/rs.3.rs-2734736/v1

**Published:** 2023-05-03

**Authors:** Beatrice Kyomugisa, Thereza Piloya Were, Joseph Rujumba, Deogratious Munube, Oriokot Lorraine, Sarah Kiguli

**Affiliations:** 1 Department of Paediatrics and Child Health, College of Health sciences, Makerere University, P.O BOX 7072 Kampala, Uganda

**Keywords:** Critically ill, Dysglycemia, hypoglycemia, hyperglycemia, outcome

## Abstract

**Introduction:**

Dysglycemia has been shown to influence outcome among critically ill children. We aimed to determine the prevalence, outcome and factors associated with dysglycemia among critically ill children aged one month to 12 years presenting to Fort Portal regional referral hospital.

**Methods:**

The study employed a descriptive, cross-sectional design for prevalence and factors associated, and longitudinal observational study design to determine the immediate outcome. Critically ill children aged one month to 12 years were systematically sampled and triaged at outpatient department using World Health Organization emergency signs. The random blood glucose was evaluated on admission and at 24 hours. Verbal and written informed consent/assent were obtained after stabilization of the study participants. Those that had hypoglycemia were given Dextrose 10% and those with hyperglycemia had no intervention.

**Results:**

Of the 384 critically ill children, dysglycemia was present in 21.7% (n = 83), of those 78.3% (n = 65) had hypoglycemia and 21.7% (n = 18) had hyperglycemia. The proportion of dysglycemia at 24 hours was 2.4% (n = 2). None of the study participants had persistent hypoglycemia at 24 hours. The cumulative mortality at 48hours was 3.6% (n = 3). At 48 hours 33.2% (n = 27) had stable blood glucose levels and were discharged from the hospital. After multiple logistic regression, obstructed breathing (AOR 0.07(0.02-0.23), inability to breastfeed/drink (AOR 2.40 (1.17-4.92) and active convulsions (AOR 0.21 (0.06-0.74), were the factors that were significantly associated with dysglycemia among critically ill children. The results will guide in the revision of policies and treatment protocols to facilitate better management of children at risk of dysglycemia nationally.

**Conclusions:**

Dysglycemia was found to affect one in five critically ill children aged one month to 12 years presenting to Fort Portal Regional Referral Hospital. Dysglycemia outcomes are good with early intervention.

## Background

Dysglycemia has been associated with increased risk of deaths most frequently in critically ill children admitted to pediatric units(1). Mortality rate in young children is increased four-to six fold when severe infectious disease is complicated by hypoglycemia (1). WHO recommends systematic screening of severely ill children for hypoglycemia, and its treatment by administering 10% dextrose (D10 %) (2). Unfortunately, treatment for hyperglycemia is controversial and, to date, no recommendations exist from pediatric professional societies regarding the management of hyperglycemia in critically ill children (3). In Africa, the prevalence of hypoglycemia among paediatric admissions has been estimated to range between 1.8% and 7.3% (4). The prevalence of hyperglycemia in tropical settings has been estimated between 2.9% and 10.9% and its presence on admission is associated with mortality (4).

In remote and resource limited settings, alterations in blood glucose levels often go undetected because of the lack of useful tools for prompt and routine blood glucose measurements (5). In most cases the presenting symptoms of hypoglycemia are often nonspecific and hypoglycemia, and more often hyperglycemia are not easily diagnosed clinically (5, 6). The prevalence and prognostic significance of hyperglycemia is less well documented and there is scarcity of data in Sub- Saharan Africa (5). In resource-limited countries, poor nutritional status, infectious diseases, delay in presentation to hospital, the use of potentially toxic herbal preparations, starvation, accidental poisoning from hypoglycemic drugs have all been found to be associated with hypoglycemia (7, 8).

A study by Mbabazi et al 2011 in ACU, Mulago reported 5.9% prevalence of hypoglycemia and 6.8% of hyperglycemia, however, the prevalence found in this study was lower than what was previously reported by other studies done in the same setting (9). In the tropics, children are particularly prone to developing hypoglycemia and hyperglycemia in a wide variety of diseases, often related to childhood malnutrition, endemic malaria, or infectious diseases (5, 10).Therefore, this study aimed to determine the prevalence, outcome and factors associated with dysglycemia among critically ill children aged one month to 12 years presenting to Fort Portal regional referral Hospital.

## Methods

### Aim of the study

To determine the prevalence, factors associated and immediate outcome of dysglycemia among critically ill children aged one month to 12 years presenting to Fort Portal regional referral hospital.

### Study design

A descriptive cross sectional study design was employed to determine the prevalence and factors associated with dysglycemia among critically ill children and with an additional assessment of the immediate outcome.

### Study setting

Triage of participants was done at OPD using WHO emergency signs and the study was conducted on Paediatric ward of Fort Portal Regional Referral Hospital. This hospital is locally known as Buhinga hospital, located in Western Uganda. It serves the entire Rwenzori Region constituting of eight districts: Kabarole, Kyenjojo, Kasese, Kamwenge, Ntoroko, Kyegegwa, Bundibugyo and Bunyangabu as well as patients from Mubende, Kibaale and parts of eastern Democratic Republic of Congo. The hospital also acts as a teaching hospital for Kampala International University (KIU), Fort Portal School of Clinical Officers, FINS nursing school, Mountains of the Moon University and CHEMIQUIP School of Clinical Officers. Additionally, it is an intern site for the doctors, nurses and pharmacists. It has an official bed capacity of 399 with an annual patient turnover of twenty-five thousand, five hundred thirty-nine patients.

The Paediatric ward has a capacity of 62 beds with an average admission of 10 to 20 patients per day. Anecdotally mortality on Paediatric ward is estimated between zero to four per day as a result of different factors. The hospital has a main laboratory that is open throughout the day and has another functional laboratory handling emergencies during the day and night. Laboratory tests routinely done are blood smears for malaria parasites, complete blood counts and hemoglobin concentration. Random blood sugars are not routinely done as a point of care test. In case random blood glucose levels cannot be ascertained, critically ill children with suspected hypoglycemia are usually treated empirically with intravenous dextrose 10%. The commonest conditions seen at the emergency department of OPD include severe malaria, severe pneumonia, severe anemia, and severe dehydration among others.

The pediatric Out Patient department of FPRRH runs throughout the entire week. The patients are seen by the medical officer, Intern doctors and clinical officers. The general paediatric clinic runs every Tuesdays in OPD department, in addition to the fore mentioned there is a pediatrician. Transfer of patients from OPD to Pediatric ward is always done immediately after clerkship and giving emergency drugs as needed. Pediatric ward has a high dependence room with three beds where critically ill children are admitted. It also has a different ward for malnourished children that belongs to Paediatric ward. Additionally, the ward has 2 government employed pediatricians and two pediatricians from KIU, 13 nurses, three medical officers, four intern doctors, two intern nurses and two cleaners. All the staff for this study were trained in triage before commencing the study. Paediatric ward and OPD were selected for the study because all critically ill patients receive their initial treatment from these two departments and the ward is covered by doctors 24 hours.

### Target population

All critically ill children aged one month to 12 years who presented to Fort Portal Regional Referral Hospital.

### Accessible population

Children aged one month to 12 years who presented to Fort Portal Regional Referral Hospital between February 2020 and April 2020 and fulfilled the study inclusion criteria.

### Actual population

These included all critically ill children who presented to Paediatric Ward and OPD of Fort Portal Regional Referral Hospital during the study period.

### Inclusion criteria

- Children aged one month to 12 years.
- Children that presented to Fort Portal Regional Referral Hospital with signs of critical illness (WHO emergency signs).
- Participants with Verbal and written informed consent by the caretakers and assent for children aged eight to 12 years.

### Exclusion criteria

Children with a known history of diabetes mellitus, infusion of dextrose containing fluids up to two hours prior to admission, those who had received Dextrose 10%, intake of steroids within 72 hours of admission, children with a known history of hypoglycemic disorder and children who die before getting treatment at admission.

### Urgent evaluation and procedures

The recruitment of study participants was done throughout the day and night. Triage was done at OPD of Fort Portal Regional Referral Hospital by the research assistants using WHO emergency signs after obtaining a verbal consent from the patients/guardians. As soon as critically ill children reached the Paediatric ward, a random blood glucose was done, the results were documented, thereafter the findings were shared with the clinical team on Paediatric ward. Those found to be hypoglycemic together with the staff on duty were given Dextrose 10%. After 20 minutes a repeat RBS was done. The participants that were found with hyperglycemia had no intervention.

If a patient declined consent standard of care was initiated immediately according to WHO protocol (1). Once the participants had stabilized on the pediatric ward, the research assistants and/or the principal investigator gave a comprehensive explanation to the parents or guardians of the selected participants, elaborated the purpose of the study, benefits, risks and thereafter request for their participation was sought. Once children eight to twelve years were stable enough and had understood the purpose of the study, they were requested to sign an assent form.

The eligible clients who agreed to participate signed a consent form by writing the initials of their names, signature and/thumb print. In case the guardian/participant could not write, in the presence of the witness a thumb print was considered. Each participant was assigned a study number and the file was labelled with a sticker to enable easy identification of the participants during the study period.

### Measurement of blood glucose level

After arrival on the ward, a verbal consent was sought if they agreed to the consent, 0. 6 mL of blood was collected by the investigators through a finger prick to measure the blood glucose concentration using Accu-Chek Performa glucometers from ROCHE Laboratories. A quality control by Accu-Chek control solutions was performed after opening a new box of test strips, when the container was left open and when the strips were subjected to low or extreme temperatures. A range outside 1.7 – 3.4 mmol/l was considered out of range. Blood glucose concentrations were recorded in mmol/l (conversion to mg/dl by multiplying by a factor of 18).

### Follow up after admission

The enrolled dysglycemic participants had a repeat RBS at 24 hours. The dysglycemic arm was also followed up at 48 hours to determine the proportion deaths and discharges. Normoglycemic study subjects were not followed up. The 24 hours was arrived at because previous studies done to evaluate inpatient dysglycemia showed that mortality was especially high 19/33 (57.6%) during the first 24 hours of admission (43)

### Data management

Data was recorded using structured pre- coded case record forms. Completed files were checked for completeness and accuracy, then stored in a lockable cabinet. All complete data was entered into an electronic database using Epidata version 3.1 software package with in-built quality control checks. The computer used for data storage and analysis was a password-protected computer and was only accessible to the PI and later, the statistician. The data was double entered and validated by the principal investigator. The final data were backed up and exported to STATA version 15 for analysis. Chi square or Fisher’s exact tests was used to compare categorical variables as appropriate. All data was treated with confidentiality.

### Data analysis

#### Descriptive statistics

To achieve objective one of this study which seeks to determine the prevalence of dysglycemia among critically ill children aged one month to 12 years presenting to Fort Portal Regional Referral Hospital, data was analyzed using descriptive analysis thereafter was summarized using proportions and percentages. Continuous variables were summarized using medians (IQR) and categorical data were summarized using proportions and percentages. To further achieve objectives 2 and 3 data was summarized using tables and figures.

#### Bivariate variables

Logistic regression was performed individually to assess the relationship between the outcome (dysglycemia) and each independent variable. The outcome was classified as: hypoglycemia: RBS < 2.5 mmol/l (45 mg/dl) in well-nourished children or 3mmol/l (< 54 mg/dl) in severely malnourished children and hyperglycemia: RBS over 8.3 mmol/l (≥150 mg/dl). Independent variables with P- value ≤ 0.2 were considered for the multivariate analysis.

#### Multivariate variables

At multivariable analysis, a multivariable logistic regression model was used to obtain factors that were independently associated with dysglycemia at p<0.05 and 95% confidence interval. Those factors with a P- value of less than 0.05 were considered to be statistically significant factors.

## RESULTS.

### Description of the study population

**Figure1:**
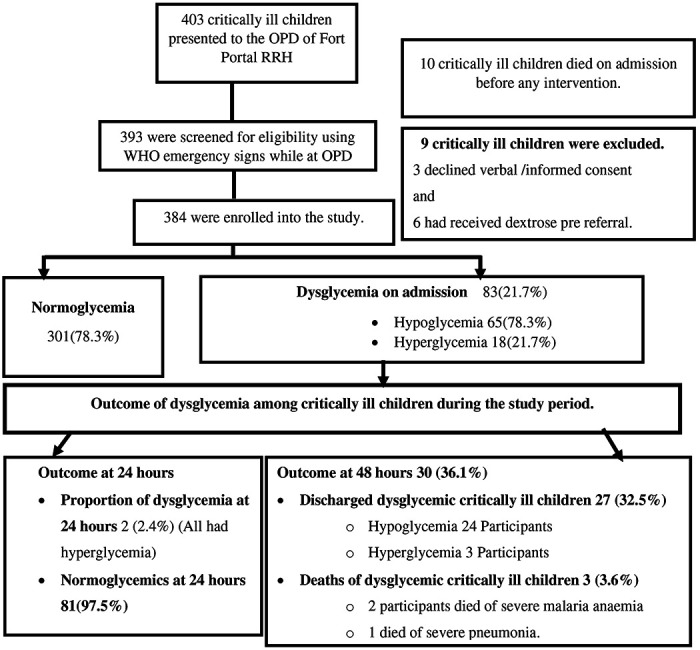
Enrolment flow chart of 384 critically ill children with dysglycemia presenting to Fort Portal Regional Referral Hospital between February 2020 to April 2020.

The study was conducted between February 2020 to April 2020. Four hundred and three (403) critically ill children presented to OPD of Fort Portal Regional Referral Hospital during this period. Of these, 393 children were screened for eligibility using WHO emergency signs while at OPD, and random blood sugars were taken off from 384 critically ill children immediately after arrival on Paediatric ward where stabilization is routinely done (Figure 1).

### Sociodemographic characteristics of 384 critically ill children presenting to Fort Portal Regional Referral Hospital between February 2020 to April 2020.

The majority of the study participants 181 of 384 (47.2%) were aged 12 to 59 months and the median age was 29.5 months (IQR (12-63 months). The male to female ratio was 1.3:1. Most critically ill children were brought to the hospital by their mothers 319 of 384 (83.1%). The highest number of caretakers had attained primary school education 163(42.4%). Details are in table 1.

### WHO emergency signs of 384 critically ill children presenting to Fort Portal Regional Referral Hospital between February 2020 to April 2020.

The commonest emergency sign among the study participants was obstructed breathing 135 (35.1%) and the least frequently observed sign was cold and blue hands 55(7.2%). Other WHO emergency signs observed in the study participants are shown in table 2.

### Clinical characteristics of the study participants

Ninety-three of (24.2%) had severe anaemia, of those 40(10.4%) had a hemoglobin level of <5g/dl. Notably, three quarters of the participants had pre-referral medication: on antimalarials were 88(23%), herbal medications 44(11.4%) and others 252(65.6%). Details are in table 3.

### Common diagnoses of 384 critically ill children presenting to Fort Portal Regional Referral Hospital between February 2020 to April 2020.

The study participants with malnutrition were 141(36.7%) and of these 96(25%) had Severe Acute Malnutrition. The other findings are shown in table 4.

### Prevalence of dysglycemia among 384 critically ill children presenting to Fort Portal Regional Referral Hospital between February 2020 to April 2020.

**Figure2:**
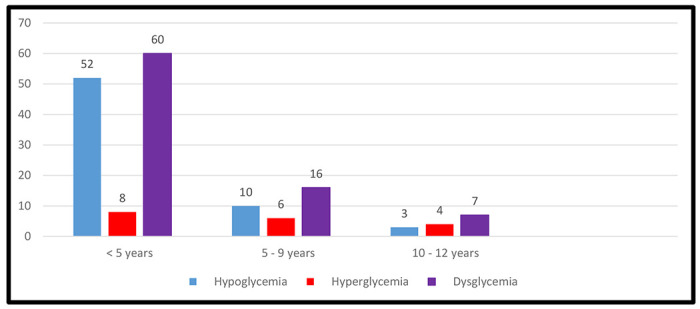
Prevalence of dysglycemia by age groups among 384 critically ill children presenting to Fort Portal Regional Referral Hospital between February 2020 to April 2020.

Of the 384 critically ill children aged one month to 12 years enrolled into this study 83(21.7%)95% CI (16.4-28.3) had dysglycemia at admission. Among these 65 of 83 (78.3%) critically ill children had hypoglycemia whereas 18 of 83 (21.7%) had hyperglycemia. The median Random blood glucose level (IQR) of the study participants on admission was 4.7 mmol/l (3.6-5.8). The majority 60(72.3%) of critically ill children with dysglycemia were under 5 years. Forty-three (51.8%) critically ill children with dysglycemia were male whereas forty (48.2%) were females. Dysglycemia increased with decreasing age. The clinical features that were independently associated with hypoglycemia were obstructed breathing (AOR = 0.06, 95% CI: 0.01-0.27) and inability to breastfeed or drink (AOR=2.28, 95% CI: 1.01- 5.15). No clinical features were associated with hyperglycemia. Details are shown in Figure 2.

### Prevalence of dysglycemia by common diagnoses among 384 critically ill children presenting to Fort Portal Regional Referral Hospital between February 2020 to April 2020.

Dysglycemia was frequent in children with acute watery diarrhea. More than a third of the study participants with dysglycemia were children with severe malnutrition 27 (32.5%) of 83 participants. Only four children with MAM presented with dysglycemia. They commonly presented with hypoglycemia. Other findings are in table 5 below.

### Bivariable analysis of clinical characteristics associated with dysglycemia.

None of the socio-demographic characteristics were significantly associated with dysglycemia at bivariable analysis. However, clinical characteristics that were significant at this level included: obstructed breathing, lethargy, active convulsions, fever, severe pneumonia, inability to feed and positive HIV status and these were considered for multivariable analysis. Other findings are shown in table 6.

### The immediate outcome of dysglycemia at 24 and 48 hours among 384 critically ill children presenting to Fort Portal Regional Referral Hospital between February 2020 to April 2020.

**Figure 3:**
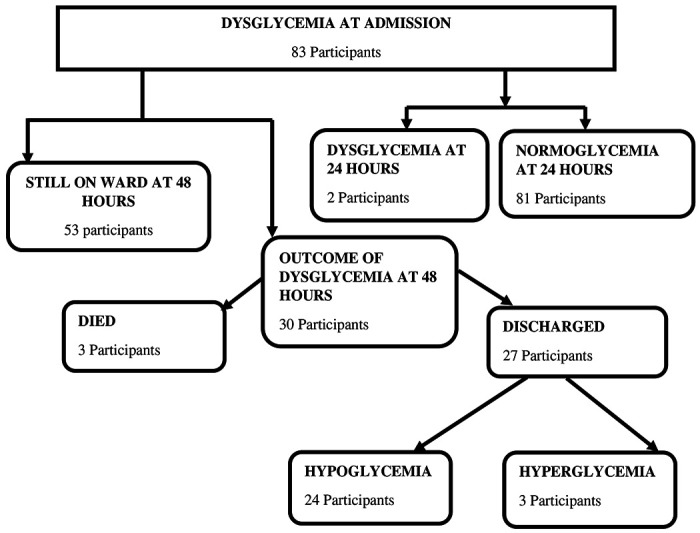
Immediate outcome of dysglycemia at 24 and 48 hours among 384 critically ill children presenting to Fort Portal Regional Referral Hospital between February 2020 to April 2020.

Of the study participants who had dysglycemia at admission only 2/83 (2.4%) remained dysglycemic (all had hyperglycemia) and the rest of the dysglycemic study participants were normoglycemic at 24 hours. Additionally, all those who had hypoglycemia at admission had normal blood glucose levels at 24 hours. Other details are in figure 1 and 3.

### Factors associated with dysglycemia at multivariable analysis

As shown in Table 7, critically ill children with inability to feed were at risk of getting dysglycemia whereas obstructive breathing and active convulsions were protective in this study.

### Multivariable analysis of clinical characteristics associated with hypoglycemia among 384 critically ill children presenting to Fort Portal Regional Referral Hospital between February 2020 to April 2020.

At multivariable analysis, logistic regression was performed by adding all the variables with p value ≤ 0.2 at bivariate level. Variables with a p value <0.05 were considered to be independently associated with dysglycemia. The factors independently associated with hypoglycemia at this level included: Obstructed breathing (AOR: 0.06(0.01-0.27), p= 0.000), inability to breastfeed or drink (AOR: 2.28(1.01-5.15), p=0.047) and hemoglobin level of less than 7g/dl (AOR: 3.22(1.01-10.24), p= 0.047). More details are shown in table 7.

**NOTE:** No factors were found to be significantly associated with hyperglycaemia in this study.

## DISCUSSION

### Introduction

This was a descriptive cross-sectional study design that set out to determine the prevalence, outcome and factors associated with dysglycemia among critically ill children aged one month to 12 years presenting to Fort Portal Regional Referral Hospital between February 2020 to April 2020.

### Prevalence of dysglycemia among critically ill children.

Overall, the prevalence of dysglycemia among critically ill children was 83(21.7%). This translates to one in five critically ill children with dysglycemia among children presenting to Fort Portal Regional Referral Hospital

The most prevalent dysglycemic anomaly was hypoglycemia of 65(78.3%) compared to hyperglycemia of 18(21.7%). The prevalence of dysglycemia increased with decreasing age. The possible reasons for the high prevalence of hypoglycemia in the study could be due to various pathophysiological mechanisms like poor oral intake, increased losses in vomiting, diarrhea and poor glucose reserve mechanisms for critically ill children that may not allow them to fast for longer periods as adults. Studies have shown that children have a limited tolerance to fasting because glycogen storage capability is limited and therefore, they are only able to maintain normal plasma glucose levels for a fasting period of 12 hours (5, 15).

These findings are comparable to those of Soro-Coulibaly et al 2018, in Côte d’Ivoire that found the prevalence of dysglycemia at 19% (12). The close similarity could be due to both studies used a descriptive, cross sectional study design, recruited critically ill children in relatively similar settings (referral hospitals) and both studies excluded critically ill children that had diabetes and those that had received dextrose within 2 hours prior to admission. Nonetheless, the observed dysglycemic rate is lower than that reported in Madagascar by Sambany et al 2013, of 34% (5). The discrepancies may be explained by variations in thresholds used to define hypoglycemia that is < 2.2 mmol/l for their study Vs < 2.5mmol/l for this study, different age ranges of above 12 years for their study participants and longer duration of five months for their study compared to three months for this study.

In contrast, Mbabazi et al 2011, observed a lower dysglycemic rate of 12.7% among critically ill children as compared to this study(2). These variations could be attributed to the shorter period of the study, different cutoffs for blood glucose values and relatively different study settings. Additionally, the later study was done in urban national referral center where children may have received therapy in previous centers as compared to this study that was conducted in a hospital that predominantly serves a rural community where children are brought from far off facilities with no standardized clinical services provided enroute to the hospital.

There are no well-defined criterion for diagnosing hyperglycemia at admission in non- diabetic critically ill children (5, 44). However, the prevalence of hyperglycemia of 21.7% in this study was much higher than that reported in the study by Soro-Coulibaly et al 2018 of 11.7% in Côte d’Ivoire (12). The differences in the findings could be because their study was conducted during the pre- COVID 19 era where patients had easy access to medical facilities before the onset of stress hyperglycemia unlike this study that was done during the COVID 19 era with stricter transport restrictions and its presumed that with this delay, critically ill children could have accessed care when the stress hyperglycemic events had already set in. However, the findings are closely similar to the 23.9% in Ethiopian study reported by Sime et al 2021. The possible explanation could be because both studies enrolled the study participants in three months and also used similar blood glucose cutoff values for hyperglycemia of > 8.3mmol/L (150mg/dL). In summary, different dysglycemic cutoff levels could have modified the results in this study in comparison to the work done by other authors(2, 5, 12).

### Outcome of dysglycemia among critically ill children.

The study evaluated the proportion of dysglycemia at 24 hours and the proportion of deaths and discharges at 48 hours. Majority 81(97.5%) of the study participants that were dysglycemic at admission had normal blood glucose levels at 24 hours compared to two (2.4%) that had persistent dysglycemia. It’s clear from this study that majority of the study participants had hypoglycemia. The fact that all hypoglycemic children were given Dextrose 10% at admission, this could have improved the outcome.

In contrast, hyperglycemia could have resolved following the treatment of the primary medical condition since its believed to be induced by critical illness (5). Only a few studies have explored blood glucose levels beyond admission and throughout hospitalization. A study by Madrid et al 2017, in Mozambique recruited critically ill children less than 15 years with malaria and had continuous blood glucose monitoring up to 72 hours but found only one child among those with hypoglycemia on admission presented subsequently with hypoglycemic episodes(45). Their findings were closely similar to the findings in this study because both used similar blood glucose cutoff values for hypoglycemia and an intervention with dextrose 10% was the standard of care basing on WHO guidelines.

The cumulative mortality was generally low among the dysglycemic children considering that critically ill children even without dysglycemia have higher mortality than this. The hospital usually reports zero to four deaths in a day during the pre COVID era, since the study was conducted in the first phase of the lockdown this could have underestimated the mortality in this study. Additionally, delayed access to care due to transport lockdown and the hierarchy of accessing travel permit to the health facilities as a result of COVID 19 pandemic could have underestimated the cumulative mortality and hindered timely access to healthcare services. Also, the fact that ten critically ill children died before admission, this may not be the true prevalence as many unregistered deaths could have occurred in the communities, thus this underrated the mortality in the study as well. Additionally, study participants with hypoglycemia had an intervention of dextrose 10%, this could have modified the outcome.

Furthermore, the turn up of patients was low due to COVID 19 pandemic therefore this gave adequate time to the health workers to provide quality care to the fewer patients. Sambany et al 2013 in Madagascar and Ameyaw et al 2014 in Ghana, reported higher mortality among critically ill children with dysglycemia of 13.2% and 13.9% respectively (5, 21). This may be because their studies were done during the pre-COVID era, had different blood glucose cutoff values and age categorization as well.

On the other hand, a smaller number of dysglycemic critically ill children were discharged from the hospital at 48 hours (Figure 3). Given that critically ill children with dysglycemia presented with WHO emergency signs in the presence of acute medical conditions this could have contributed to delayed discharge from the hospital. Studies by Wintergerst et al, 2006 in California and Sambany et al, 2013 in Madagascar, found dysglycemic children had a longer length of hospital stay (5, 28). In the current study and in previously mentioned studies majority of the dysglycemic study participants were under 5 years, thus with the background evidence of reduced immune system in the setting of critical illness, this could have lengthened the period of hospital stay.

### 5.4 Factors associated with dysglycemia among the study participants

In general inability to feed increased the risk of dysglycemia whereas active convulsions and obstructed breathing were protective. No factor was found to be independently associated with hyperglycemia.

Critically ill children with inability to breastfeed and drink were 2.4 times more likely to get dysglycemia. The children that had longer duration of illness of above seven days and prolonged fasting of greater than 2 days were more predisposed to dysglycemia (Table 7). The possible cause for the findings could be because these children are too ill to feed orally in the presence of loss of consciousness and anorexia induced by critical illness. Furthermore, prolonged fasting depletes the glycogen stores and heightens the risk of dysglycemia.

In addition, studies have shown that long travel to the health facility deprives critically ill children a chance to be fed along the way thus prolonging the fasting period(5, 15). In this study 46% of the participants were from other neighboring districts. The findings of this study are higher than those of Sambany et al, 2013 that found 10.4% had this symptom(5). This could be because they excluded many patients in their study and also had a 24 hour break every after 48 hours of data collection, this could have underestimated the prevalence in their study.

Obstructed breathing was protective for dysglycemia in this study. The reason behind this could be because obstructed breathing is taken as a sign of critical illness in the community and children are brought to the health facility timely. Additionally, it is a common practice for children with obstructed breathing to have a nasal gastric tube inserted because of the feared risk of aspiration pneumonia. However, there are very few studies that have used this emergency sign at triage. A prospective observational study by Mawji et al in Kenya 2018 found obstructed breathing was not statistically significant in children aged two to 60 months seeking treatment for an acute illness(46). Nevertheless, they recruited critically ill children below the age of five years unlike in this study where study participants were recruited every day and included critically ill children from one month to twelve years.

Also, critically ill children with convulsions were less likely to be dysglycemic in this study. This could be because it’s a common practice for children with convulsions to receive dextrose from health worker’s pre-referral because convulsions are considered a sign of hypoglycemia and as well as an indicator of loss of consciousness. The dysglycemic findings of 5(6.0%) are much lower than those reported by Sambany et al, 2013 and Barennes et al 2016 that found 31.6% and 51.4% respectively had dysglycemia with convulsions (5, 15). These variations could be due to the different thresholds that were used to classify dysglycemia.

### Strengths of the study.

This is one of a few studies that have used the WHO cutoffs for hypoglycemia in both well-nourished and malnourished critically ill children. Furthermore, it’s the first study in our setting that has been conducted from a peripheral health facility to evaluate the two disorders simultaneously (hypoglycemia and hyperglycemia).

### Limitations of the study.

1. The study did not specifically screen participants for Diabetes mellitus and Diabetic ketoacidosis, however, the history and clinical evaluation helped to exclude their clinical presentation for the children that had hyperglycemia.
2. Additionally, the study was conducted during the first phase of the lockdown due to COVID 19 pandemic (March to April 2020), this therefore may have underestimated the mortality and the prevalence rate of dysglycemia.
3. Furthermore, due to vehicle restrictions especially motorcycles which are the commonest mode of transport to facilities, access to healthcare was limited and thus fewer children from distant places were reaching the referral centers like Fort Portal Hospital, this therefore, could have underrated the prevalence of dysglycemia during the study period.
4. Likewise, children from distant locations from the hospital, had higher possibilities of delay, often associated with poor health outcomes, and these might have been underrepresented in the study leading to the low prevalence of dysglycemia and a low cumulative mortality.
5. Also, comparisons with other studies are difficult due to the different thresholds used for hypoglycemia and hyperglycemia. In the current study this was overcome by use of the WHO cutoff values for hypoglycemia and also by using of International Society for Pediatric and Adolescent Diabetes (ISPAD) guidelines for the cutoff of hyperglycemia.
6. Lastly the study was unable to assess the long term outcome of dysglycemia among critically ill children.
  On the other hand, limitations 2, 3, and 4 were difficult to overcome because we had no control on the government lockdown guidelines.

### Conclusions

Dysglycemia is a common disorder affecting one in five critically ill children aged one month to 12 years presenting to Fort Portal Regional Referral Hospital. Dysglycemia outcomes are good with early intervention among critically ill children.

### Recommendations

1. Critically ill children with dysglycemia who present with inability to feed should be prioritized for clinical care in emergency settings by the clinical team. The interpretation of the findings of obstructed breathing and active convulsions should have interpreted with caution given that the study was done in an era of COVID19 pandemic where transport restrictions were tight.
2. A larger prospective study among critically ill children would be necessary to determine the long-term outcome of dysglycemia.
3. The availability of bedside diagnostic tools to measure blood sugar levels may accelerate clinical decision-making in the management of critically ill children with dysglycemia in resource-constrained settings. Every critically ill child with emergency signs should have blood glucose levels monitored regularly during their hospital stay by the clinicians.

## Figures and Tables

**Table 1:** Baseline characteristics of 384 critically ill children presenting to Fort Portal Regional Referral Hospital between February 2020 to April 2020.

Variables		Frequency (N=384)	Percentage (%)
Age of the child	1-11 months	85	22.1
	12-59 months	181	47.2
	60-119 months	85	22.1
	120-144 months	33	8.6

Gender	Male	217	56.6
	Female	167	43.4

Next of kin	Mother	319	83.1
	Father	40	10.4
	Others	25	6.5

Education level of the caretaker	None	142	30.0
	Primary	163	42.4
	Secondary or higher	79	20.6

Referrals	Yes	153	39.9
	No	231	60.1

Household income per month	≤ 27USD (≤100,000 Ushs)	238	69.8
	>27USD (>100,000 Ushs)	103	30.2

District of origin	Kabarole	210	54.7
	Others	174	45.3

**Table 2:** WHO emergency signs at triage of 384 critically ill children presenting to Fort Portal Regional Referral Hospital between February 2020 to April 2020.

Variables		Frequency (N=384)	Percentage (%)
Obstructed breathing	Yes	135	35.1
	No	249	65.0

Central cyanosis	Yes	50	13.0
	No	334	87.0

Rapid and weak pulse	Yes	55	14.3
	No	329	85.7

Cold and blue hands	Yes	28	7.2
	No	356	92.8

Capillary refill > 3 sees	Yes	83	21.6
	No	301	78.4

Lethargy/ loss of consciousness	Yes	61	15.9
	No	323	84.1

Severe dehydration	Yes	77	20.0
	No	307	80.0

Active convulsions	Yes	69	18.0
	No	315	82.0

Note: Participants could present with more than one WHO emergency sign

**Table 3:** Clinical characteristics of 384 critically ill children presenting to Fort Portal Regional Referral Hospital between February 2020 to April 2020.

Variables		Frequency (N=384)	Percentage (%)
Fever	Yes	255	66.4
	No	129	33.6

Inability to breastfeed or drink	Yes	109	28.3
	No	275	71.7

Cough	Yes	266	69.2
	No	118	30.8

Difficult breathing	Yes	166	43.2
	No	218	56.8

Vomiting	Yes	169	44.0
	No	215	56.0

Diarrhea	Yes	128	33.3
	No	256	66.7

Pre-referral medication#	Yes	286	74.5
	No	98	25.5

Duration of illness	< 6 days	272	70.8
	≥7 days	112	29.2

Duration of last meal	≤ 1 day	48	12.5
	2-7 days	278	72.4
	> 7 days	58	15.1

Nutritional status	Normal	243	63.2
	MAM	45	11.8
	SAM	96	25

Blood slide for malaria	Positive	91	23.7
	Negative	293	76.3

Hemoglobin level*	<7 g/dl (severe anemia)	93	24.2
	7-9.9 g/dl (Moderate anemia)	102	26.5
	10-10.9 g/dl (Mild anemia)	63	16.4
	>10.9 g/dl (Normal)	126	32.9

HIV status	Negative	276	71.9
	Positive	16	4.1
	Not known	92	24.0

# Participants had used more than one medication on admission.

* WHO grading of anemia

**Table 4:** Common medical diagnoses of 384 critically ill children presenting to Fort Portal Regional Referral Hospital between February 2020 to April 2020.

Admission diagnoses	Frequency	Percentage (%)
Severe pneumonia	142	37.0
Malnutrition (MAM/SAM)	141	36.7
Acute watery diarrhea	128	33.3
Severe anaemia	93	24.2
Severe malaria	91	23.6
Suspected septicemia	69	17.9
HIV/AIDS	16	4.1
Sickle Cell Anemia	16	4.1

Note: Some study participants had more than one diagnosis.

**Table 5:** Distribution of dysglycemia by common diagnoses among 384 critically ill children presenting to Fort Portal Regional Referral Hospital between February 2020 to April 2020.

Diagnosis	Frequency (N=83)	Percentage (%)
Acute watery diarrhea	36	43.4
Malnutrition (MAM/SAM)	31	37.3
Severe anemia	25	30.1
Severe pneumonia	16	19.3
Severe malaria	16	19.3
Suspected septicemia	13	15.7
HIV/AIDS	7	8.4
Sickle Cell Anemia	6	7.2

**Table 6:** Bivariable analysis of clinical characteristics associated with dysglycemia among 384 critically ill children presenting to Fort Portal Regional Referral Hospital between February 2020 to April 2020.

Variables		Normoglycemia (N=301)	Dysglycemia (N=83)	COR 95% CI	P value
**WHO emergency signs**					

Obstructed breathing	Yes	130(43.4)	5(6.0)	0.08(0.33,0.22)	**0.000**
	No	171(56.6)	78(94.0)	1.00	

Lethargy/ loss of consciousness	Yes	56(18.5)	5(6.0)	0.29(0.11,0.74)	**0.010**
	No	245(81.5)	78(94.0)	1.00	

Active convulsions	Yes	64(21.2)	5(6.0)	0.24(0.09, 0.62)	**0.003**
	No	237(78.8)	78(94.0)	1.00	
**History, examination findings and common diagnoses.**					

Fever	Yes	211(70.2)	44(53.0)	0.49(0.29,0.81)	**0.005**
	No	90(29.8)	39(47.0)	1,00	

Inability to breastfeed or drink	Yes	75(24.8)	34(41.0)	2.14(1.28,3.57)	**0.003**
	No	226(75.2)	49(59.0)	1.00	

Duration of sickness	<6 days	207(68.8)	65(78.3)	1.00	
	≥7 days	94(31.2)	18(21.7)	0.61(0.34,1.08)	0.092

Duration of last meal					
1 day and below		34(11.3)	14(16.9)	1.00	
2-7 days		223(74.1)	55(66.2)	0.59(0.30,1.19)	0.145
>7 days		44(14.6)	14(16.9)	0.77(0.32,1.84)	0.559

Watery diarrhea	Yes	92(30.6)	36(43.4)	1.76(1.07,289)	**0.027**
	No	209(69.4)	47(56.6)	1.00	

Severe pneumonia	Yes	126(41.7)	16(19.3)	0.34(0.19,0.61)	**0.000**
	No	175(58.3)	67(80.7)	1.00	

Nutritional status	Normal	191(63.6)	52(62.7)	1.00	
	Moderate	41(13.6)	4(4.8)	0.37(0.13,1.07)	**0.067**
	Severe	69(22.9)	27(32.5)	1.47(0.86,2.53)	0.161

HIV status	Negative	224(74.5)	52(62.7)	1.00	
	Positive	9(3.0)	7(8.4)	3.37(1.19,9.45)	0.021
	Not known	68(2.5)	24(28.9)	1.46(0.84,2.56)	0.183

**Table 7:** Logistic regression for factors independently associated with dysglycemia of 384 critically ill children presenting to Fort Portal RRH between February 2020 to April 2020.

Variables		COR 95% CI	P value	AOR 95% CI	P value
Obstructed breathing	Yes	0.09(0.34,0.22,18)	0.000	**0.07(0.02,0.23)**	0.000
	No	1.00		1.00	

Active convulsions	Yes	0.24(0.09, 0.62)	0.003	0.21(0.06,0.74)	0.016
	No	1.00		1.00	

Fever	Yes	0.49(0.29,0.81)	0.005	0.58(0.29,1.18)	0.136
	No	1,00		1.00	

Lethargy/Loss of consciousness	Yes	0.29(0.11,0.74)	0.010	0.39(0.10,1.56)	0.188
	No	1.00		1.00	

Inability to breastfeed or drink	Yes	2.14(1.28,3.57)	0.003	2.40(1.17,4.92)	0.017
	No	1.00		1.00	

Watery diarrhea	Yes	1.76(1.07,2.89)	0.027	1.78(0.82,3.89)	0.144
	No	1.00		1.00	

Nutritional status*	Normal	1.00		1.00	
	Moderate	0.37(0.13,1.07)	0.067	0.24(0.07,0.83)	0.024
	Severe	1.47(0.86,2.53)	0.161	1.45(0.68,3.06)	0.335

COR; Crude Odds Ratio, AOR; Adjusted Odds Ratio, CI; Confidence.

* Weight for height or height for age or weight for age or BMI for age (WHO growth charts 2006).

## Data Availability

The original data set will be made available by the corresponding author upon reasonable request.

## List of abbreviations

ACU: Acute Care Unit
AIDS: Acquired Immune Deficiency Syndrome
ART: Anti- Retroviral Therapy
BG: Blood Glucose
DM: Diabetes Mellitus
FRRH: Fort Portal Regional Referral Hospital
HIV: Human Immunodeficiency Virus
ICU: Intensive Care Unit
IMCI: Integrated Management of Childhood Illness
ISPAD: International Society for Pediatric and Adolescents Diabetes
OPD: Out Patient Department
PFC: Pediatric Filter Clinic
Pre- DM: Pre-Diabetes Mellitus
RBS: Random Blood Glucose
RR: Relative Risk
SAM: Severe Acute Malnutrition
SHG: Spontaneous Hypoglycemia
WHO: World Health Organization
